# Accurate *Ab Initio* and Template-Based Prediction of Short Intrinsically-Disordered Regions by Bidirectional Recurrent Neural Networks Trained on Large-Scale Datasets

**DOI:** 10.3390/ijms160819868

**Published:** 2015-08-21

**Authors:** Viola Volpato, Badr Alshomrani, Gianluca Pollastri

**Affiliations:** 1 School of Computer Science, University College Dublin, Belfield, Dublin 4, Ireland; E-Mails: volpato.viola@gmail.com (V.V.); badr.alshomrani@ucdconnect.ie (B.A.); 2 Adaptive and Complex Systems Laboratory, University College Dublin, Belfield, Dublin 4, Ireland

**Keywords:** protein disorder, BRNN, MobiDB, PDB

## Abstract

Intrinsically-disordered regions lack a well-defined 3D structure, but play key roles in determining the function of many proteins. Although predictors of disorder have been shown to achieve relatively high rates of correct classification of these segments, improvements over the the years have been slow, and accurate methods are needed that are capable of accommodating the ever-increasing amount of structurally-determined protein sequences to try to boost predictive performances. In this paper, we propose a predictor for short disordered regions based on bidirectional recurrent neural networks and tested by rigorous five-fold cross-validation on a large, non-redundant dataset collected from MobiDB, a new comprehensive source of protein disorder annotations. The system exploits sequence and structural information in the forms of frequency profiles, predicted secondary structure and solvent accessibility and direct disorder annotations from homologous protein structures (templates) deposited in the Protein Data Bank. The contributions of sequence, structure and homology information result in large improvements in predictive accuracy. Additionally, the large scale of the training set leads to low false positive rates, making our systems a robust and efficient way to address high-throughput disorder prediction.

## 1. Introduction

The structure-function paradigm has for a long time maintained that functions of proteins are related to their three-dimensional structure [1] and that, in general, a primary sequence of amino acids is associated with a regular three-dimensional structure. In fact, it is now clear that many proteins only partly fold to a native regular structure or are normally completely unfolded [2,3,4,5,6,7,8]. While the function for some proteins depends on the permanent disordered state of a portion of their structure, it has also emerged that in numerous other proteins, short disordered regions can undergo a disorder-to-order transition after binding to other macromolecules [9]. The lack of a definite ordered three-dimensional structure has been associated with the major functional advantages for these proteins in structural flexibility and plasticity [10]. These proteins, which can recognise multiple target molecules with high specificity and low affinity [6,11], are indeed rich in functional sites, such as for binding to other proteins, membranes and nucleic acids [12,13], and for cellular localization signals, alternative splicing and post-translational modifications [14,15]. Their relevant roles in many biological processes, as in regulation, signalling and cell cycle control [2,16], mean that they are involved in many functions related to diseases, including cancer, cardiovascular disease, diabetes, neurodegenerative diseases and amyloidoses [12,17,18]. Moreover, disorder annotations have been shown to improve protein function prediction [19]. The importance of intrinsically-disordered proteins in functional proteomics [19,20] and the collection of protein disorder sequences in comprehensive databases thanks to a continually increasing number of structurally-determined proteins foster the development of accurate computational methods able to include as much information as possible for the prediction and identification of these regions.

Proteins or segments of the protein chain that do not adopt a stable structure are known, respectively, as intrinsically-disordered proteins (IDPs) or regions (DRs) [3,21] and can affect various levels of protein structure [22]. Computational approaches have made progress over the last decade and a half in predicting disordered regions in proteins. These methods can be roughly classified into two categories. Firstly, some methods rely on simple rules based on amino acids propensities [23,24,25,26]. Other methods use (sometimes quite sophisticated) machine learning algorithms trained on datasets of known protein disordered regions [18,23,27,28,29,30,31,32,33,34]. In all cases, homology to known disordered proteins (templates) can be introduced into the predictive pipeline [35,36], similarly to the case of the prediction of other structural features [37,38,39,40], sometimes leading to considerable improvements in accuracy. A good review of disorder prediction methods can be found in [41].

In spite of this progress, many problems remain. Among them, the fact that disorder is rarer than order leads to a skewed classification problem in which good recall of disordered regions often leads to very high numbers of false positives. The definition itself of disorder is the subject of some contention, with manually curated sets, e.g., DISPROT [42], representing only a tiny fraction of all proteins of known structure, which is a substantial hindrance for the more sophisticated machine learning algorithms that tend to be data greedy and have been shown to steadily improve as data sizes grow [43]. Partly because of this, there have been recent efforts to standardise the definition of disorder, leading to automated, large-scale datasets of disordered regions [44] directly extracted from the Protein Data Bank [45,46]. In this article, we rely on one such resource, MobiDB, to train new predictors of disorder.

## 2. Results and Discussion

In this work, we investigate whether informative combinations of sequence, structural features and direct annotation from templates significantly improve disorder prediction over sequence-based inputs and the extent to which sequence similarity between the query and the templates contributes to predictive accuracy.

We train a standard feed-forward NN and BRNN using the full combination of information and a fixed window size of 21 residues that was shown to be the best combination on a preliminary training set. We show that our best predictors, trained with the richest input coding systems, achieve high-quality performances, especially at low FPRs.

**Figure 1:**
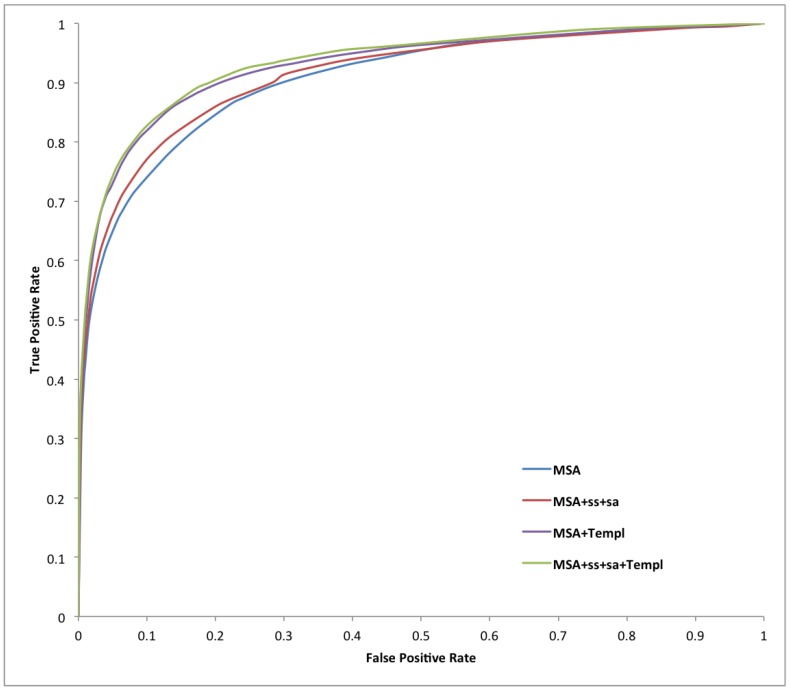
Receiver-operating characteristic curves for all of our systems on X-ray test set data.

**Figure 2:**
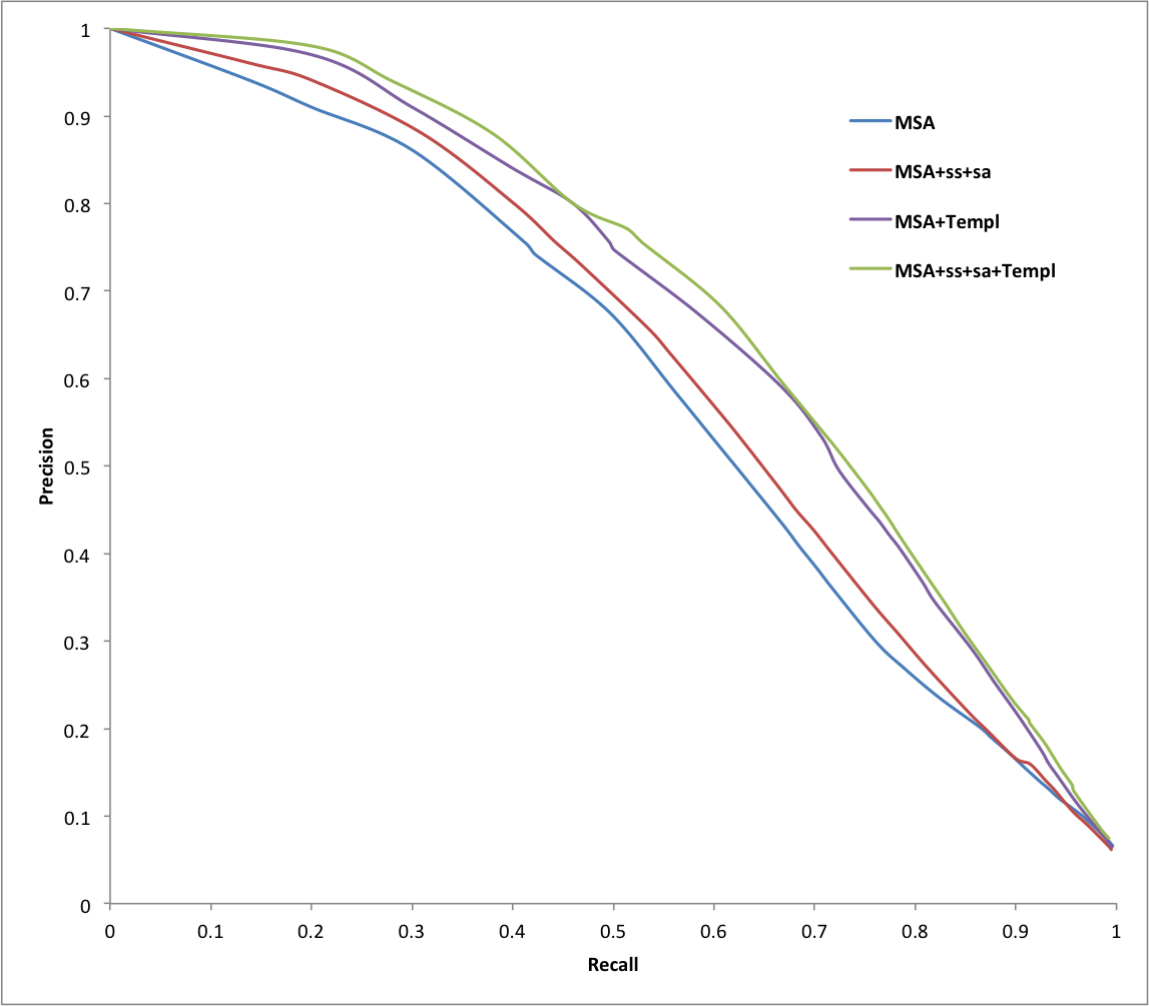
Precision-recall curves for all of our systems on X-ray test set data.

**Figure 3:**
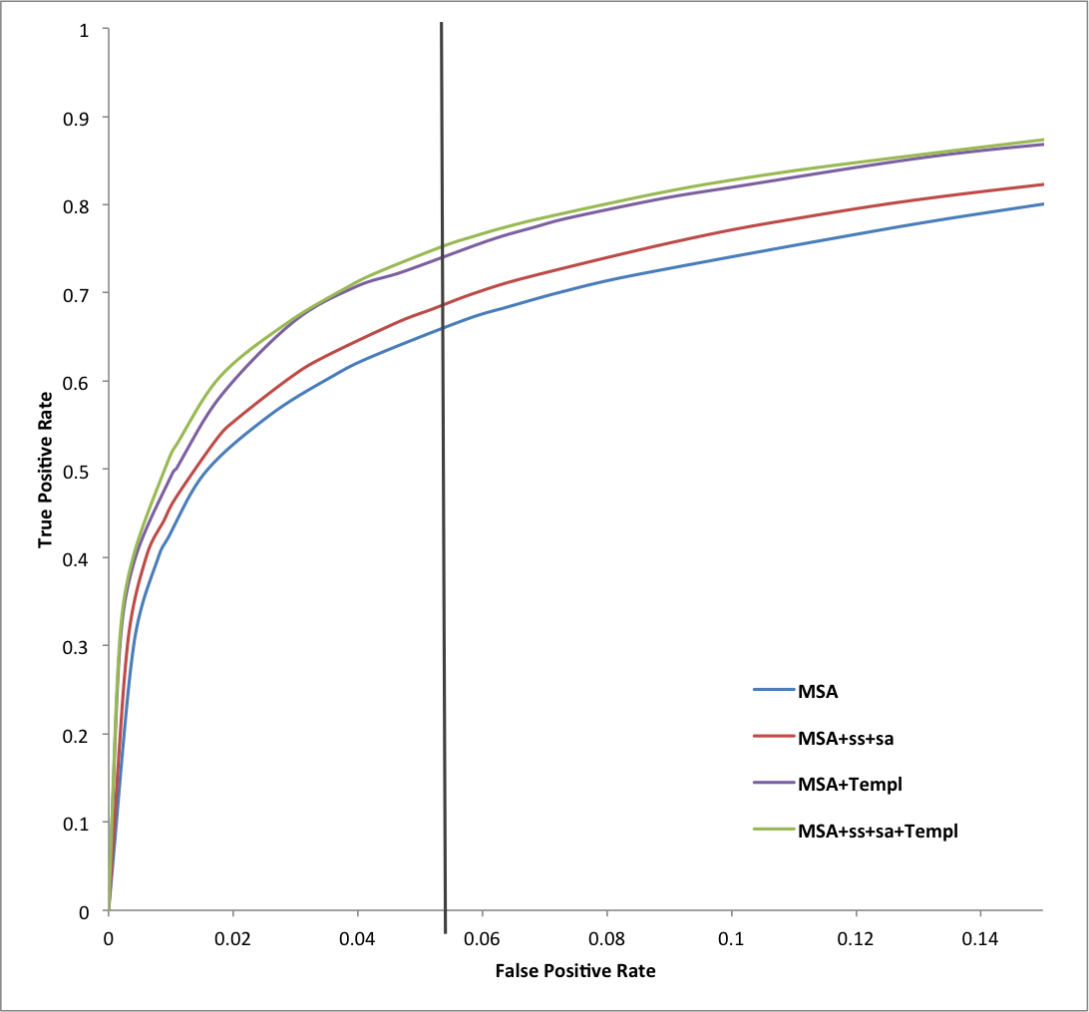
Receiver-operating characteristic curve for X-ray test set data showing the FPR in the region from 0% to 14% false positives for all of our methods. The vertical line represents the decision thresholds corresponding to a predicted 5% FPR.

Table 1, Table 2 and Table 3 report a summary of the results on both training and test sets using different combinations of evolutionary inputs, structural features predicted by Porter (secondary structure) and PaleAle (solvent accessibility), as well as weighted information from disordered templates as input to the BRNNs and to a standard NN containing 20 hidden units and taking as input a window of 21 residues. The number of false positives is limited, to prevent drawing false conclusions on the prevalence of disorder, without affecting the predictors’ sensitivity. This is shown in Figure 1 and Figure 2, and especially in Figure 3, which contains a typical ROC curve plot on the test set focused on low FPRs. A stringent 5% expected FPR threshold selected on the training dataset for our best predictor (MSA-SS-SA-Templ) gives the test set 0.751 sensitivity and 0.462 Prec at 5.5% FPR (black line on the plot). The very high specificity of our systems makes them potentially useful in high-throughput analysis of protein disorder, where the main goal is to create predictors producing a minimum number of false positives [47].

For the comparative experiments of different input information integrated in our systems, all performance measures reported in Table 1 and Table 2 clearly indicate that structural information encoded into templates improves classification performances with respect to the use of sequence information alone. An MCC increment to 0.605 (MSA-Templ) from 0.432 (MSA) and from 0.570 (MSA-SS-SA) to 0.615 (MSA-SS-SA-Templ) is evident. However, when sequence information, secondary structure and solvent accessibility predictions and templates are combined, a further improvement is observed over the other cases in terms of MCC.

**Table 1:** Result of Sensitivity (Sens), Specificity (Spec) , precision (Prec), accuracy (Acc) and Matthews’ correlation coefficient (MCC) on training sets for all of our systems.

Predictor	Sens	Spec	Prec	Acc	MCC
MSA	0.867	0.847	0.268	0.857	0.430
MSA-SS-SA	0.534	0.983	0.671	0.732	0.568
MSA-Templ	0.584	0.983	0.692	0.783	0.615
MSA-SS-SA-Templ	0.606	0.982	0.684	0.794	0.622
NN	0.698	0.877	0.220	0.632	0.377

These results show that introducing homology-based information is generally advantageous. The minimal improvements observed instead when adding predicted secondary structure and solvent accessibility might be caused by the noisy nature of the secondary structure and solvent accessibility predictions. Another explanation might be related to the fact that, as recently reported in [1], a majority of disordered proteins have some residual transient secondary structures that are required for function. The use of predicted secondary structure in the inputs might skew early learning of the models and later prevent inclusion of the effect of useful, but subtler information, such as the MSA.

We also compare our predictors to state-of-the-art methods based on Sensitivity (Sens), Specificity (Spec) and AUC (ROC). These methods, among which CSpritz, ESpritz, MULTICOM, PONDR-FIT, Disopred and IUPred, have been tested and evaluated on an X-ray test set of 569 structures as reported in [34]. Although this comparison (Table 4) has to be taken with caution because of the use of different test sets (our test set is two times larger than the one used by the other methods), it shows that all of our variants present high specificity and clearly outperform the methods used for comparison in terms of AUC (ROC) values.

**Table 2:** Results of Sensitivity (Sens), Specificity (Spec), Precision (Prec), Accuracy (Acc) and MCC on the test set for all of our systems.

Predictor	Sens	Spec	Prec	Acc	MCC
MSA	0.818	0.871	0.284	0.845	0.432
MSA-SS-SA	0.535	0.982	0.657	0.738	0.570
MSA-Templ	0.582	0.982	0.676	0.782	0.605
MSA-SS-SA-Templ	0.603	0.981	0.672	0.792	0.615
NN	0.693	0.847	0.218	0.628	0.372

**Table 3:** Results of AUC_ROC and AUC_PR on the test set for all systems.

Predictor	AUC_ROC	AUC_PR
MSA	0.919	0.634
MSA-SS-SA	0.913	0.614
MSA-Templ	0.920	0.658
MSA-SS-SA-Templ	0.925	0.661
NN	0.914	0.617

## 3. Experimental Section

### 3.1. Bidirectional Recurrent Neural Networks

In [31,34], bidirectional recurrent neural networks (BRNNs) [48] have already been successful at predicting protein disorder based only on information at the sequence level. Similar BRNNs have been applied to other prediction problems, e.g., secondary structure [49] and solvent accessibility [37]. In protein sequences, the properties under investigation at position *j* depend on the local context surrounding *j* and on parts of the sequence that are distant from *j*, but close in 3D space. In contrast to regular feed-forward neural networks that use sliding windows of a predetermined size and cannot learn information from the entire sequence, BRNNs can learn this information implicitly from the surrounding local context through the recursive dynamics of the network itself. BRNNs also benefit from having paths of variable length between inputs and outputs, which may grow as deep as the length of the input sequence.

To learn the mapping from the space I of inputs $I=(i_{1},...,i_{N})$ to the space O of outputs $O=(o_{1},...,o_{N})$ (where *N* is the sequence length), a BRNN exploits the N-terminal and the C-terminal sequence contexts using three separate neural networks that take the form:

$$\begin{array}{c} o_{j}=N^{\left(O\right)}\left(i_{j},h_{j}^{\left(F\right)},h_{j}^{\left(B\right)}\right)\\ h_{j}^{\left(F\right)}=N^{\left(F\right)}\left(i_{j},h_{j-1}^{\left(F\right)},\cdots ,h_{j-S}^{\left(F\right)}\right)\\ h_{j}^{\left(B\right)}=N^{\left(B\right)}\left(i_{j},h_{j+1}^{\left(B\right)},\cdots ,h_{j+S}^{\left(B\right)}\right) \end{array}$$

where each $i_{j}\in I$ is the input coding of the amino acid in position *j* and $h_{j}^{\left(F\right)}$ and $h_{j}^{\left(B\right)}$ are forward and backward chains of hidden vectors with $h_{0}^{\left(F\right)}$ = $h_{N+1}^{\left(B\right)}$ = 0. The output, forward transition and backward transition functions (respectively $N^{\left(O\right)}$, $N^{\left(F\right)}$ and $N^{\left(B\right)}$) are implemented by three two-layered feed-forward neural networks [37]. As in [49], a top layer filter is included in the network that takes ‘semi-global’ information as input from the bottom layer. The *i*-th input to this second network includes the first-layer predictions in position *i* augmented by first stage predictions averaged over multiple contiguous windows. That is, if $c_{j1},...,c_{jm}$ are the outputs in position *j* of the first stage network corresponding to estimated probabilities of vector component *j* being in class *m*, the input to the second stage network in position *j* is the array $I_{j}$:

(1) $$\begin{array}{c} I_{i}=(c_{j1},\cdots ,c_{jm},\\ \sum_{h=k_{-p}-w}^{k_{-p}+w}c_{h1},\cdots ,\sum_{h=k_{-p}-w}^{k_{-p}+w}c_{hm},\\ \cdots \\ \sum_{h=k_{p}-w}^{k_{p}+w}c_{h1},\cdots ,\sum_{h=k_{p}-w}^{k_{p}+w}c_{hm}) \end{array}$$

where $k_{f}=j+f\left(2w+1\right)$, 2w+1 is the size of the window over which first stage predictions are averaged and 2p+1 is the number of windows considered. In the tests, we use *w* = 7 and *p* = 10. This means that 18 contiguous, non-overlapping windows of 18 residues each are considered, *i.e.*, first stage outputs between position *j* − 162 and *j* + 162, for a total of 324 contiguous residues, are taken into account to generate the input to the filtering network in position *j*. This input contains a total of 19*m* real numbers: *m* representing the *m*-class output of the first stage in position *j*; 18*m* representing the *m*-class outputs of the first stage averaged over each of the 18 windows.

### 3.2. Input Encoding

The input *i* associated with the *j*-th residue contains evolutionary information, secondary structure and solvent accessibility information, as well as homology information derived from PDB templates:

$$\begin{array}{c} i_{j}=\left(i_{j}^{\left(E\right)},i_{j}^{\left(S\right)},i_{j}^{\left(A\right)},i_{j}^{\left(T\right)}\right)\\ where:\\ i_{j}^{\left(E\right)}=\left(i_{j,1}^{\left(E\right)},...,i_{j,e}^{\left(E\right)}\right)\\ i_{j}^{\left(S\right)}=\left(i_{j,1}^{\left(S\right)},...,i_{j,e}^{\left(S\right)}\right)\\ i_{j}^{\left(A\right)}=\left(i_{j,1}^{\left(A\right)},...,i_{j,e}^{\left(A\right)}\right)\\ i_{j}^{\left(T\right)}=\left(i_{j,1}^{\left(T\right)},...,i_{j,e}^{\left(T\right)}\right) \end{array}$$

where *e* = 21 units are dedicated to evolutionary information, *s* = 3 to secondary structure, *a* = 4 to solvent accessibility and *t* = 3 to homology information. Evolutionary information is incorporated in the form of frequency profiles derived from alignments of multiple homologous sequences (MSA). As in our previous works [50,51], for each protein, a profile is built as follows: the *k*-th residue in a protein is encoded as a sequence of 21 real numbers in which each number is the frequency of one of the 20 amino acids in the *k*-th column of the MSA plus an additional real number used to represent the frequency of gaps in the *k*-th column. Sequence alignments are extracted from uniref90 [52] from February 2010 containing 6,464,895 sequences. The alignments are generated by three runs of PSI-BLAST [53] with expectation of a random hit set to 0.001 and the threshold for inclusion into the PSSM set to $10^{-10}$ [54].

The additional information provided by secondary structure as predicted by the Porter server [49] is encoded in three additional inputs (helix, strand and coil), and information by solvent accessibility predicted by the PaleAle server [37] is encoded in four additional inputs (completely buried, partly buried, partly exposed and exposed).

To generate homology information from known structures (templates) for a protein, we run two rounds of PSI-BLAST against the version of the redundancy-reduced uniref90 described above, with the same parameters as for the MSA search. We then run a third round of PSI-BLAST against the PDB using the PSSM generated in the first two rounds. We further remove from each set of hits found all those with sequence similarity exceeding 95% over the whole query. For each residue *j*-th in a query sequence *i*, a vector *N* + 1 terms long, where N=2, that is the number of classes predicted (ordered and disordered), is calculated as:

(2) $$\vec{T_{j}}=\left[\frac{\sum_{p=1}^{p}\vec{V_{p,j}}I_{p}^{3}}{\sum_{p=1}^{p}I_{p}^{3}},score_{i}\right]$$

where $I_{p}$ is the identity between the query *i* and template *j* and $\vec{V_{p,j}}$ is a vector of *N* units encoding the ordered and disordered state of the *j*-th residue in the *p*-th template as follow:

(3) $$\begin{array}{c} O=(1,0)\\ D=(0,1) \end{array}$$

where *O* represent the ordered state and *D* the disordered state of the *j*-th residue. The N+1-th element in the vector $\vec{T_{j}}$ measures the significance of the information stored in the vector and is computed as the average identity, weighed by the cubed identity. That is:

(4) $$score_{i}=\frac{\sum_{p=1}^{p}I_{p}I_{p}^{3}}{\sum_{p=1}^{p}I_{p}^{3}}=\frac{\sum_{p=1}^{p}I_{p}^{4}}{\sum_{p=1}^{p}I_{p}^{3}}$$

The contribution of low-similarity templates is reduced compared to high-similarity templates, when available, by taking the cube of the identity scores. For instance, templates that are more closely related to the protein are weighed more than more remote ones, *i.e.*, a 90% identity template is weighed two orders of magnitude more than a 20% one [37]. Because all indices are residue-based, in case of templates covering fragments of a protein or in case of a lack of templates, the section of the input for this residue with template information is left blank, and predictions will be based only on the sequence and predicted structural features. This means that there is no need to employ different predictors for different types of input.

It should be noted that this information is given to the neural network as input and not directly combined into a prediction. The weight and importance of templates alongside all of the other information about a protein is automatically determined during training.

For the comparative experiments, we build four final systems that we refer to as MSA (evolutionary information), MSA-SS-SA (evolutionary, secondary structure and solvent accessibility information), MSA-SS-SA-Templ (evolutionary, secondary structure, solvent accessibility information and templates) and MSA-Templ (evolutionary information and templates).

Table 4 shows the number of inputs for a given residue in each system.

**Table 4:** The number of inputs per component for a given residue in each of the six systems. SS, secondary structure; SA, solvent accessibility; Templ, template.

System	Input Component				
	**e**	**s**	**a**	**t**	**Total**
**MSA**	21	0	0	0	21
**MSA-SS-SA**	21	3	4	0	28
**MSA-Templ**	21	0	0	3	24
**MSA-SS-SA-Templ**	21	3	4	3	31

For the sake of comparison, we also train a standard feed-forward neural network (NN) in the same conditions as the BRNNs. In preliminary testing on a smaller set, we determined the optimal window size to be 21 amino acids.

## 4. Training/Testing Datasets

To train and test the performance of our predictors, we collect our dataset from MobiDB (release of June 2012). Since we aim to train our method on a large dataset of high quality short regions, only sequences with structure obtained by X-ray crystallography with a resolution <2.0 Å, chains of a length >50 and containing at least three, but a maximum of 30 consecutive disordered residues are selected. Homology reduction is performed within the whole set, removing the instance of lesser quality of any sequences showing more than 30% mutual identity. The final dataset of short disordered regions consists of 6415 proteins with 1,612,256 residues of which 6.03% are disordered.

To perform five-fold cross-validation, the set is split into five subsets with an equal number of chains. As a result of the large dimension of the subsets, the number of residues in each fold is also approximately the same, and the distribution of disordered/ordered residues closely mimics that of the whole set, thus increasing the robustness of the experiment. This would be more difficult to achieve in the case of the analysis of sequences with long disordered regions that are five-times less than sequences with short disordered segments. To compare the performances of our predictors to state-of-the-art methods, the CASP10 data are downloaded from the official website (URL: http://predictioncenter.org/casp10/). Note that residues marked as “*x*” in CASP10 were not considered in the analysis as in the CASP10 assessment.

### 4.1. Training Protocol, Ensembling

Five two-stage BRNN models are trained by minimising the cross-entropy error between the output and target probability distributions, using gradient descent with no momentum term or weight decay. The gradient is computed using backpropagation through the structure algorithm. BRNN models are trained independently and ensemble averaged to build the final predictor. For each training, we save and ensemble average the nine best performing networks (on testing). An ensemble of nine such copies for all five models is used (45 models in total), because improvements between 1.5% and 2% are achieved with respect to an ensemble of five models in total. We use 500 batch blocks per training set. Thus, the weights are updated 500-times per epoch. Two thousand epochs of training are performed for each model. The training set is shuffled at each epoch. The learning rate is halved every time we do not observe a reduction of the error for more than 500 epochs.

### 4.2. Measuring Performances

To assess the performance of our predictors, we use similar measures as in CASP10 [55]. For all of our predictors, after the probability decision thresholds are determined on the corresponding training sets, binary measures are calculated as follows:

(5) $$\begin{array}{cc} & SE=\frac{TP}{\left(TP+FN\right)}\\ & SP=\frac{TN}{\left(TN+FP\right)}\\ & Prec=\frac{TP}{\left(TP+FP\right)}\\ & Acc=\frac{1}{2}\left(\frac{TP}{\left(TP+FN\right)}+\frac{TN}{\left(TN+FP\right)}\right)\\ & MCC=\frac{TP\cdot TN-FP\cdot FN}{\sqrt{\left(TP+FP)(TN+FP)(TP+FN)(TN+FN\right)}} \end{array}$$

where SE is the sensitivity, also referred to as the recall; SP is the specificity; Prec is the precision; Acc is the balanced accuracy; MCC is Matthews’ correlation coefficient [56]; TN (true negatives) is the number of correctly-classified ordered residues, FN (false negatives) is the number of disordered residues incorrectly classified as ordered; FP (false positives) is the number of ordered residues incorrectly classified as disordered; TP (true positives) is the number of correctly-classified disordered residues. Prec, Acc and MCC are considered in [55] to be the most appropriate measures for the evaluation of the disorder data, as they place more weight on the prediction of the minority class (disordered residues). However, MCC is considered a more balanced measure than Prec and Acc. MCC does not reward overprediction of disorder as much as Acc and is not completely insensitive to the prediction of the dominant class (ordered state) as Prec. Thus, MCC is seen as better suited to identify classifiers with higher precision and to estimate the quality of disorder predictors.

The second type of measures evaluate the accuracy of identifying disorder by assigning per-residue disorder confidence scores. These are the receiver operating characteristic (ROC) and the precision recall (PR) curve analyses. For a set of probability thresholds (from zero to one), a residue is considered as a positive example (disordered) if its predicted probability is equal to or greater than the threshold value. An ROC curve is a monotonic function describing the balance between sensitivity (or true positive rate, TPR) and one-specificity (or false positive rates, FPR). The area under this curve (AUC-ROC) is an aggregate measure of the overall quality and is particularly useful as an indicator of algorithm robustness. A value of one corresponds to a perfect classifier, while 0.5 indicates a random prediction. For the first time in CASP10, the PR curve is introduced, because it is particularly suitable for statistical evaluations on disproportional datasets in contrast to the ROC curves that overestimate the performance of predictors on the imbalanced data [55]. The PR curves are conceptually similar to the ROC curves, but differ in that they are plotted in the recall precision coordinates and are not necessarily monotonous. As in ROC curve analysis, the area under the PR curve, AUC-PR, is indicative of the classifier’s accuracy, with a value of one corresponding to a perfect predictor [55].

### 4.3. Applicability of Templates

**Figure 4:**
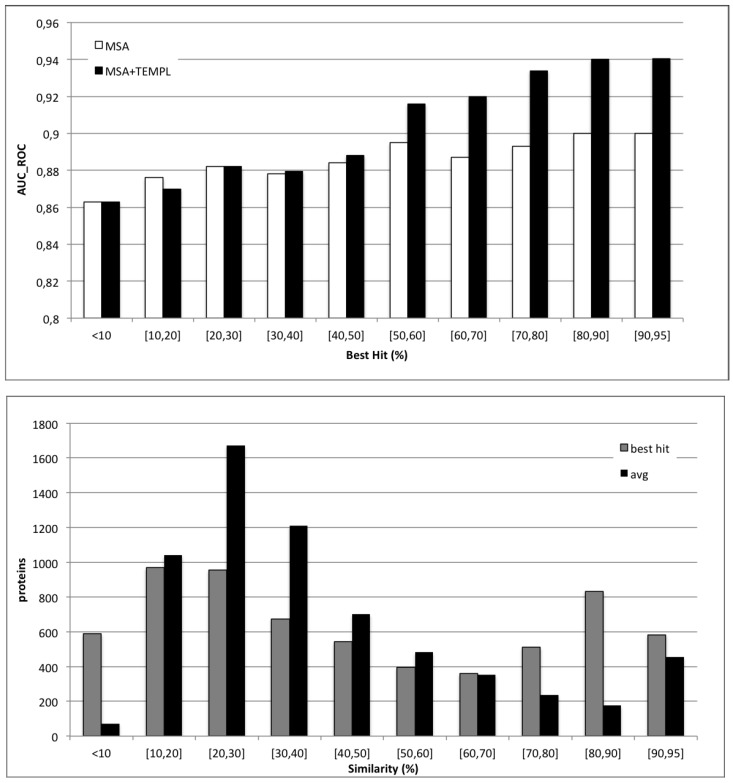
Distribution of the area under the ROC curve as a function of sequence identity of the examples to the best template that could be found. Black bars represent template-based results (MSA-Templ) and white bars sequence-based results (MSA) (**top**); The number of proteins with templates within given ranges of sequence identity. Black bins represent average sequence identity; grey bins identity to the best template (**bottom**).

To further explore the relationship between performance and structural information from templates, in Figure 4, the histogram of the AUC is plotted as a function of sequence identity between the query and its best template, and the numbers of proteins in different classes of sequence identity to the best template are also reported. In order to measure the effect of sequence identity to the best template (that is, how much the quality of templates affects the performances of the system), we subdivide the results into bins of 10% sequence identity. Two different sets of results in this binned version are computed: results including homology (MSA-Templ) reported as black bars; and sequence-based results (MSA) reported as grey bars. Similarly to the case of protein function, thresholds to transfer disorder annotation tend to be higher than those for transferring structure. The MSA-Templ results are clearly superior to the MSA ones (sequence-based) for values of sequence identity to the best template above 50% and only marginally so in the 30% to 50% range. The distribution of templates is also represented in Figure 4. Overall, template-based results are more accurate than sequence-based ones in approximately two thirds of the cases in our experiments.

### 4.4. CASP10 Results

In order to fully compare our method to the state-of-the-art methods, we also use data from the CASP10 experiment. Table 5 shows the top five (out of 28) groups plus our systems ranked by MCC. Our predictors are tested on all 94 targets and on 85 targets that have less than 30% sequence identity to our training protein sequences in order to test our methods on a completely independent set. For the template-based version, we tested our predictors both on high quality templates, with up to 95% sequence identity with each target, and on lower quality, with up to 50% sequence identity (see Figure 4 for the sequence identity-based distribution of the first template available for each target). Our results suggest a consistently good performance of our predictors, especially when taking into account that some of the top five are meta-servers. Even in the absence of close homologous proteins, our methods still achieve good results for all performance measures. In addition, at a decision threshold of 0.5, which is the one used in CASP10 to compare different predictors, all FPRs are lower than 1% (data not shown). At 5% FPRs, our predictors achieve sensitivity values around and higher than 0.6, ranking similarly to the best predictor DISOPRED3, as reported in [55].

As in the CASP10 assessment, we also verify the accuracy of prediction of the internal disordered regions, which is expected to be a harder problem, as unstructured residues are more abundant at the termini. To do so, we repeat our tests after trimming 10 residues from each terminal of the targets. Table 6 shows evidence that our best method fares well, with an MCC value of 0.480 compared to an MCC value slightly over 0.4 for DISOPRED3. Moreover, the new AUC scores for all of our predictors are only slightly lower than the AUC of our versions on full sequences. This can be considered as an indication that our algorithms are almost equally accurate in assigning disorder probabilities to residues inside the proteins and at the termini.

**Table 5:** Results for Sens, Spec and AUC on X-ray test sets for all of our systems and the top-ranking methods as reported in Walsh *et al.* (2012) [34].

Predictor	Sen	Spec	AUC_ROC
MSA-SS-SA-Templ	0.603	0.981	0.925
MSA-Templ	0.582	0.982	0.920
MSA-SS-SA	0.535	0.982	0.913
MSA	0.818	0.871	0.919
CSpritz	0.796	0.850	0.899
ESpritz	0.773	0.856	0.891
SSpritzP	0.765	0.870	0.889
ESpritzP	0.775	0.853	0.888
MULTICOM	0.820	0.804	0.888
PONDR-FIT	0.692	0.867	0.861
IUPred(short)	0.540	0.949	0.847
Disopred	0.565	0.939	0.839

**Table 6:** Performances on the termini-trimmed targets obtained by all our versions and by top five CASP10 methods, derived from Monastyrskyy *et al.* (2014) [55], according to the MCC and AUC scores.

Predictor	MCC	AUC_ROC
MSA-SS-SA + templ95_94_internal	0.480	0.873
MSA-SS-SA + templ95_85_internal	0.476	0.869
MSA-templ95_94_internal	0.434	0.865
MSA-templ95_85_internal	0.426	0860
MSA-SS-SA + templ50_85_internal	0.405	0.851
DISOPRED3	0.405	0.850
MSA-SS-SA + templ50_94_internal	0.400	0.857
MSA-SS-SA_94	0.393	0.845
MSA-SS-SA_85	0.389	0.840
MSA_94	0.377	0.831
Prods-CNF	0.375	0.865
Biomine_dr_mixed	0.370	0.850
MSA_85	0.368	0.821
MSA-templ50_94_internal	0.345	0.833
MSA-templ50_85_internal	0.334	0.831
DisMeta	0.325	0.625
Biomine_dr_pdb_c	0.315	0.850

## 5. Conclusions

Intrinsically-disordered regions playing key roles in numerous proteins are important for our understanding of many cellular mechanisms, thus of protein structure and function. The demand for accurate predictors of disordered regions can be met by fully exploiting the large number of experimentally-characterized disordered proteins now available in comprehensive databases. This is especially valid for short disordered sequences that are over-represented compared to long disordered segments.

Here, we propose accurate and specialised predictors for short disordered sequences powered by BRNNs that can accommodate this vast amount of biological data. The predictors are trained on a curated, non-redundant and very large dataset that extensively covers this type of disorder flavour. The enriched input coding, which integrates evolutionary information, predicted secondary structure and solvent accessibility and direct disorder annotation from homologous proteins deposited in PDB, makes our systems very accurate in predicting disorder whilst minimising false positives. This might make our system applicable to high-throughput problems where the number of false positives has to be kept at low levels. Our best predictor also shows high performances in terms of MCC and Prec values when compared to state-of-the-art methods and provides competitive predictions as evaluated on datasets with low-sequence similarity. It is also capable of equally predicting disorder on termini and internal regions of proteins, as demonstrated on CASP10 targets. Therefore, we expect that, if we assume that disorder is conserved across members of the same family, the ever-increasing amount of structurally-determined protein sequences and the growth of disorder annotation databases will certainly improve our predictive performances over time. Additionally, even in the presence of low evolutionary conservation, our way to design models proved to be strong in building methods intrinsically able to recognize patterns of amino acids that show high sequence variability [50,51]. In the case of disordered regions, our predictors are likely already capable of automatically recognizing specific physico-chemical properties that have been described for short and long disordered regions.

The predictor described in this work is freely available to academic users at the address: http://distillf.ucd.ie/newpunch/. Multiple queries in FASTA format, up to 64 kbytes of total input per batch, can be submitted to the server, which will return the results as a single email and/or web page if no email is provided.
